# Case fatality inequalities of critically ill COVID-19 patients according to patient-, hospital- and region-related factors: a French nationwide study

**DOI:** 10.1186/s13613-021-00915-4

**Published:** 2021-08-19

**Authors:** Antoine Guillon, Emeline Laurent, Antoine Duclos, Lucile Godillon, Pierre-François Dequin, Nelly Agrinier, Antoine Kimmoun, Leslie Grammatico-Guillon

**Affiliations:** 1 Intensive Care Unit, Tours University Hospital, Research Center for Respiratory Diseases, INSERM U1100, University of Tours, Tours, France; 2 Epidemiology Unit EpiDcliC, Service of Public Health, Tours University Hospital, 2 Bd Tonnellé, 37044 Tours Cedex 9, France; 3 Research Unit EA1075 (Education Ethique et Santé), University of Tours, Tours, France; 4 Research on Healthcare Performance Lab (RESHAPE) INSERM U1290, University of Claude Bernard Lyon 1, Lyon, France; 5 Health Data Department, Hospices Civils de Lyon, Lyon, France; 6 CHRU-Nancy, INSERM, Université de Lorraine, CIC, Epidémiologie Clinique, 54000 Nancy, France; 7 Teaching Hospital of Nancy, Intensive Care Unit, University of Lorraine, Nancy, France; 8 MAVIVH, INSERM U1259; University of Tours, Tours, France

**Keywords:** COVID-19, ICU, Determinant of lethal outcomes

## Abstract

**Background:**

The COVID-19 sanitary crisis inflicted different challenges regarding the reorganization of the human and logistic resources, particularly in intensive care unit (ICU). Interdependence between regional pandemic burden and individual outcome remains unknown. The study aimed to assess the association between ICU bed occupancy and case fatality rate of critically ill COVID-19 patients.

**Methods:**

A cross-sectional study was performed in France, using the national hospital discharge database from March to May, 2020. All patients admitted to ICU for COVID-19 were included. Case fatality was described according to: (i) patient’s characteristics (age, sex, comorbid conditions, ICU interventions); (ii) hospital’s characteristics (baseline ICU experience assessed by the number of ICU stays in 2019, number of ICU physicians per bed), and (iii) the regional outbreak-related profiles (workload indicator based on ICU bed occupancy). The determinants of lethal outcome were identified using a logistic regression model.

**Results:**

14,513 COVID-19 patients were admitted to ICU; 4256 died (29.3%), with important regional inequalities in case fatality (from 17.6 to 33.5%). Older age, multimorbidity and clinical severity were associated with higher mortality, as well as a lower baseline ICU experience of the health structure. Regions with more than 10 days with ≥ 75% of ICU occupancy by COVID-19 patients experienced an excess of mortality (up to adjusted OR = 2.2 [1.9–2.6] for region with the highest occupancy rate of ICU beds).

**Conclusions:**

The regions with the highest burden of care in ICU were associated with up to 2.2-fold increase of death rate.

**Supplementary Information:**

The online version contains supplementary material available at 10.1186/s13613-021-00915-4.

## Introduction

The COVID-19 has been recently called a *syndemic* [1, 2]. This concept aims to describe how COVID-19 spreads with pre-existing conditions, but also how it is driven by larger political, economic, and social factors [1, 3]. To reconfigure conventional understanding of COVID-19 mortality, we should integrate healthcare contexts in which it occurred. In France, from March to May 2020, the COVID-19 outbreak massively affected the country, but with substantial differences in regional incidence ranges. Metropolitan France (i.e., on the European continent) is divided into 13 administrative regions, sharing the same universal healthcare system. The French healthcare system is a universal service for every citizen, irrespective of wealth, age or social status, made up of a fully integrated network of public and private hospitals, and other medical service providers.

The SARS-CoV2 virus, equally virulent intrinsically in these 13 regions, brought the similar clinical presentation, suggesting that each critically ill patient in France had theoretically the same risk of surviving or dying. However, a more nuanced approach is needed because this threat had inflicted different challenges regarding the reorganization of the human and logistic resources, particularly in intensive care unit (ICU) hospitalizations for advanced monitoring and live support. High burden on the healthcare system has already been observed in France in responding to terrorist attack but on a short period and in a restricted area [4]. This time and without any precedent, the healthcare system has been overwhelmed in some French regions, the number of critical care beds and dedicated health workers being a critical factor [5]. The ICU bed occupancy, defined as the number of critically ill COVID-19 patients divided by the number of ICU beds, has emerged as a dynamic workload indicator to assess the ICU burden. However, the number of ICU beds was difficult to establish because there was a constant flow of ICU reorganization (i.e., expansion of ICU beds, novel ICU locations within hospital or in pop-up hospital). Thus, the ICU occupancy was defined based on the ICU bed resource that was present before the pandemic [6]. Whether the outcome of critically ill patient was affected by the ICU occupancy is unknown. Mortality rate was higher within ICUs located in Paris and the northeast regions but the direct influence of logistic and organizational aspects, along with the incidence rates, on patient outcomes has not been proven [5]. We need to take a more *holistic* approach including the environmental factors such as hospital structures and region-related factors, to improve the comprehension of outcome of critically ill COVID-19 patients. The objective of the study was to assess the impact of ICU bed occupancy on case fatality rate of critically ill COVID-19 patients.

## Methods

### Study design and data collection

A cross-sectional study, using medico-administrative data from the exhaustive French hospital discharge database (HDD) (*Programme de Médicalisation des Systèmes d’Information—PMSI*) was performed with the data available on the national dedicated French secured platform (*Agence Technique de l’Information sur l’Hospitalisation—ATIH)*. In France, it is mandatory to report data from all hospital stays at public and private hospitals. All information from reported hospitalizations is stored in the HDD as medical codes [International Classification of Diseases, Tenth Revision (ICD-10)]. All patients are assigned a unique identification number, allowing the same individual to be followed over time. The encrypted anonymized patient number, allowing the estimation of pre-existing comorbidities based on their 2-year anteriority (PMSI 2018-19) [7–9].

Patients were included according to the following inclusion criteria: adults (≥ 18 years old), admitted to one French ICU (public or private sector), between March, 1st and May 31st 2020, with at least one night spent in ICU (including ICU and step-down units), and at least one ICD-10 diagnosis code of COVID-19 whatever their position in the hospital stay resume (Additional files 1, 2: Supplementary data).

Socio-demographic and clinical data related to these patients were extracted from the French HDD. Hospital and regional data were selected in the administrative section of the HDD from the *Statistique Annuelle des Etablissements (SAE)* available through the *ATIH* secured platform [6]. In case of transfer, the region retained for the analyses was the one with the longest ICU stay.

### Variables of interest

Outcomes of COVID-19 patients hospitalized in ICU in France were analyzed according to three levels of variables:

Level 1—Patient characteristics: vital status at the end of the hospital stay (primary outcome), sociodemographics characteristics (age, sex), comorbid conditions (Additional files 1, 2), SAPS II (Simplified Acute Physiology Score II) at the first admission in the ICU (missing data if score < 5, *n* = 281), and specific care supports: mechanical ventilation (invasive or non-invasive, with/without prone position), renal replacement therapy, extra-corporeal membrane oxygenation, vasoactive treatments.

Level 2—Hospital characteristics: type of hospital (teaching or regional hospitals, local or private or mixed sector facilities), baseline ICU experience (approximated by the number of adult ICU stays in the hospital in 2019—before the pandemic), and number of ICU physicians per healthcare facility (expressed as the ratio of ICU physicians in 2019 divided by the number of ICU beds in the hospital).

Level 3—Regional characteristics: workload indicator based on the French indicator of ICU burden defined as the number of days with 75% or more of the ICU beds at baseline (before the COVID crisis) occupied by COVID-19 patients in the region, estimated per day over the study period.

### Statistical methods

The continuous variables were described by their mean ± standard deviation (SD), whereas the qualitative variables were described with effectives and percentages. A description of the variables of interest was performed for the overall population, and then stratified according to: (i) the region of the longest COVID-19 ICU stay and (ii) the vital status at the end of the last COVID-19 ICU stay during the study period.

To identify the risk factors associated with the ICU case fatality, first, bivariate analyses were performed with the variables of interest above. Second, a logistic regression model was carried out, including variables with *p* < 0.2 in bivariate analysis, as well as variables considered as clinically relevant. Third, a descending stepwise process was used to select the final model of logistic regression, including all the statistically significant variables at the threshold *p* < 0.05. All variables, including those related to the patient, hospital and region, were included in the same model. Analyses were performed using SAS Enterprise Guide 71 64-bit (SAS Institute Inc., Cary, NC, USA), version available on the *ATIH* website at the moment of the analyses.

### Ethical approval

No nominative, sensitive or personal data of patients have been collected. Our study involved the reuse of already recorded and anonymized data. The study falls within the scope of the French Reference Methodology MR-005 (declaration 2205437 v 0, August 22nd, 2018, subscripted by the Teaching Hospital of Tours), which require neither information nor consent of the included individuals. This study was consequently registered with the French Data Protection Board (*CNIL* MR-005 number #2018160620).

## Results

**Fig. 1:**
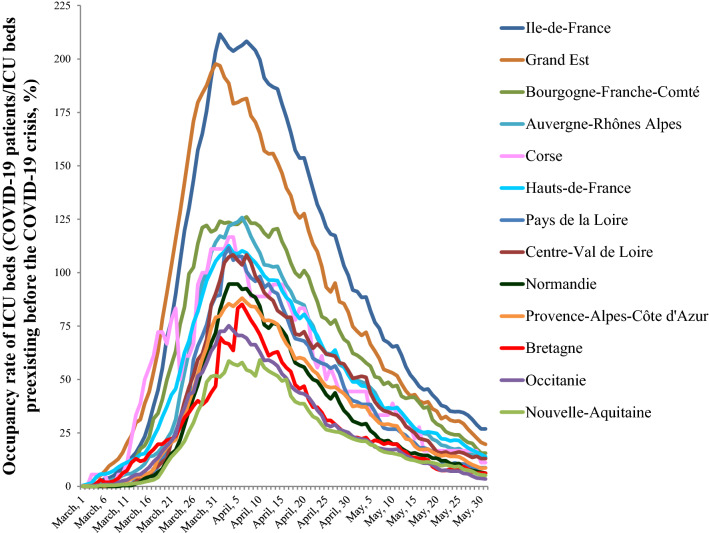
Evolution of occupancy rate of ICU beds by COVID-19 patients, according to the French administrative region. The number of beds refers to ICU beds pre-existing before the COVID-19 crisis

**Fig. 2:**
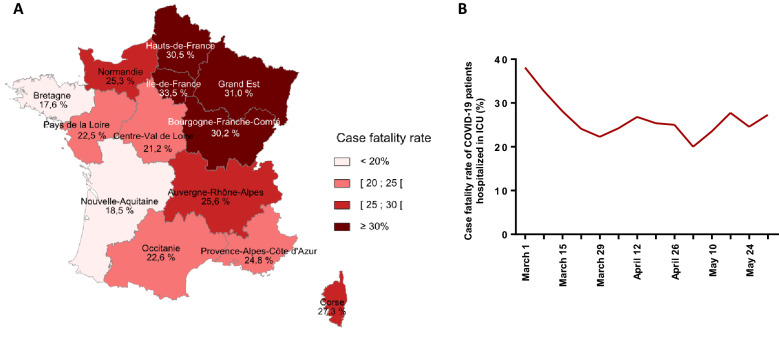
Case fatality rate of COVID-19 patients admitted to ICU in France, March 1st to May 31st, 2020. Case fatality rate of COVID-19 patients according to: **A** the French administrative region, **B** the date of admission

We identified 14,513 adult patients with at least one ICU stay attributed to COVID-19, among 58,033 total ICU patients from March, 1rst to May, 31rst 2020. Critically ill COVID-19 patients represented 25% of all ICU-hospitalized patients in France during this period. However, important differences were observed at the regional level (Table 1): French administrative regions localized on the West part of the country (*Bretagne, Pays de la Loire, Nouvelle Aquitaine*) had 10 to 12% COVID-19 patients admitted to ICUs whereas this ratio reached 39 to 42% in North-East region (*Grand Est*) and in Paris with the surrounding area (*Ile-de-France*). Moreover, the occupancy of ICU beds by COVID-19 patients varied widely over time (Fig. 1). A simultaneous peak around April 1st was observed in all regions but with large differences in intensity and duration among them, whereas the patients’ characteristics were broadly similar across the country (Table 1). The patients were mainly male (78%; sex ratio 2.5), with a mean age of 63 ± 13 years old and 80.9% had at least one comorbid condition, the most frequent being high blood pressure, chronic heart diseases and diabetes mellitus (Table 1). The initial SAPS II was 39.5 (± 17) in France with little variability between regions (lowest value 36.0 ± 15.3; highest value 41.5 ± 17.4). The specific care supports were invasive mechanical ventilation for 68.1% of the cases and vasopressors for 58.8% (Table 1). Eventually, 4,256 COVID-19 patients died during their hospital stay, representing a 29.3% case fatality rate, varying from 17.6% to 33.5% according to the administrative region (Fig. 2A) and from 20.0% to 38.1% according to the period (Fig. 2B).

**Table 1:** Characteristics of COVID-19 patients admitted in the ICU, according to the French administrative region, March, 1st to May, 31st 2020

Population 2017 (*n*)	Overall population		*Auvergne-Rhône-Alpes*		*Bourgogne-Franche-Comté*		*Bretagne*		*Centre-Val de Loire*		*Corse*		*Grand Est*	
	64,639,133		7,949,036		2,810,551		3,318,904		2,577,191		334,938		5,549,586	
Patient in ICU—all causes (*n*)	58,033		6942		2235		2166		1831		202		5500	
Critically ill COVID-19 patients in ICU (*n*, %)	14,513	25.0%	1358	19.6%	580	26.0%	256	11.8%	410	22.4%	44	21.8%	2130	38.7%
Age class (*n*, %)														
< 65 y.o	7145	49.2%	521	38.4%	213	36.7%	110	43.0%	182	44.4%	14	31.8%	928	43.6%
65–79 y.o	6280	43.3%	698	51.4%	299	51.6%	129	50.4%	196	47.8%	25	56.8%	1066	50.0%
≥ 80 y.o	1088	7.5%	139	10.2%	68	11.7%	17	6.6%	32	7.8%	5	11.4%	136	6.4%
Age (mean ± s.d.)	63 ± 13		65.9 ± 12.4		66.9 ± 11.5		63.4 ± 13.3		64.2 ± 12.6		67.2 ± 13.2		64.4 ± 11.9	
Sex ratio (M/F)	2.5		2.7		2.3		2.4		2.3		2.4		2.6	
SAPS II (mean ± s.d.)	39.47 ± 17		38.85 ± 16.1		41.46 ± 15.6		37.69 ± 16.6		37.73 ± 15.9		39.91 ± 15.3		42.09 ± 17.4	
Comorbidities (*n*, %)	11,739	80.9%	1132	83.4%	494	85.2%	196	76.6%	356	86.8%	33	75.0%	1782	83.7%
High blood pressure	7131	49.1%	695	51.2%	293	50.5%	122	47.7%	229	55.9%	16	36.4%	1158	54.4%
Chronic heart disease	4971	34.3%	545	40.1%	243	41.9%	84	32.8%	157	38.3%	14	31.8%	893	41.9%
Diabetes	4487	30.9%	447	32.9%	189	32.6%	73	28.5%	150	36.6%	8	18.2%	667	31.3%
Obesity	3549	24.5%	328	24.2%	139	24.0%	59	23.0%	129	31.5%	5	11.4%	563	26.4%
Cancer	3078	21.2%	370	27.2%	174	30.0%	64	25.0%	128	31.2%	14	31.8%	567	26.6%
Chronic renal disease	2352	16.2%	310	22.8%	93	16.0%	46	18.0%	84	20.5%	4	9.1%	448	21.0%
Chronic pulmonary disease	1913	13.2%	218	16.1%	96	16.6%	34	13.3%	75	18.3%	6	13.6%	319	15.0%
Neurological disease	1120	7.7%	166	12.2%	64	11.0%	12	4.7%	33	8.0%	2	4.5%	204	9.6%
Chronic liver disease	1046	7.2%	102	7.5%	41	7.1%	16	6.3%	35	8.5%	1	2.3%	184	8.6%
ICU specific care supports (*n*, %)														
Central venous catheter	7407	51.0%	697	51.3%	258	44.5%	137	53.5%	215	52.4%	25	56.8%	1389	65.2%
Continuous hemodynamic monitoring	7601	52.4%	774	57.0%	225	38.8%	114	44.5%	238	58.0%	30	68.2%	1319	61.9%
Vasoactive treatment*	8528	58.8%	772	56.8%	367	63.3%	142	55.5%	230	56.1%	16	36.4%	1438	67.5%
Renal replacement therapy	2165	14.9%	212	15.6%	64	11.0%	31	12.1%	49	12.0%	3	6.8%	259	12.2%
ECMO	587	4.0%	25	1.8%	13	2.2%	6	2.3%	13	3.2%	2	4.5%	82	3.8%
Mechanical ventilation** (n, %)														
Non invasive/high flow oxygenotherapy	6546	45.1%	664	48.9%	241	41.6%	105	41.0%	191	46.6%	16	36.4%	824	38.7%
Invasive	9885	68.1%	866	63.8%	430	74.1%	165	64.5%	276	67.3%	21	47.7%	1,652	77.6%
Invasive with prone position	5534	38.1%	534	39.3%	257	44.3%	101	39.5%	165	40.2%	14	31.8%	967	45.4%
Death (*n*, %)	4256	29.3%	347	25.6%	175	30.2%	45	17.6%	87	21.2%	12	27.3%	660	31.0%

	*Hauts-de-France*		*Ile-de-France*		*Normandie*		*Nouvelle-Aquitaine*		*Occitanie*		*Pays de la Loire*		*Provence-Alpes-Côte D’azur*	
Population 2017 (*n*)	6,003,815		12,174,880		3,330,478		5,956,039		5,845,209		3,757,600		5,030,906	
Patient in ICU—all causes (*n*)	5464		13,620		2451		4833		5142		2964		4683	
Critically ill COVID-19 patients in ICU (*n*, %)	1333	24.4%	5650	41.5%	387	15.8%	503	10.4%	667	13.0%	355	12.0%	840	17.9%
Age class (*n*, %)														
< 65 y.o	624	46.8%	3321	58.8%	177	45.7%	248	49.3%	286	42.9%	170	47.9%	351	41.8%
65–79 y.o	579	43.4%	2016	35.7%	180	46.5%	212	42.1%	318	47.7%	166	46.8%	396	47.1%
≥ 80 y.o	130	9.8%	313	5.5%	30	7.8%	43	8.5%	63	9.4%	19	5.4%	93	11.1%
Age (mean ± s.d.)	63.9 ± 12.9		61 ± 13.2		63.6 ± 12.2		63.7 ± 12.6		64.2 ± 13.3		62.4 ± 13.2		65.3 ± 13.2	
Sex ratio (M/F)	2.3		2.6		2.4		2.5		2.3		2.7		2.3	
SAPS II (mean ± s.d.)	41.79 ± 18.6		38.76 ± 17.2		36.81 ± 14.8		39.5 ± 18.2		37.19 ± 16.1		35.95 ± 15.3		39.27 ± 15.6	
Comorbidities (*n*, %)	1151	86.3%	4364	77.2%	326	84.2%	403	80.1%	537	80.5%	285	80.3%	680	81.0%
High blood pressure	740	55.5%	2571	45.5%	207	53.5%	236	46.9%	306	45.9%	154	43.4%	404	48.1%
Chronic heart disease	524	39.3%	1505	26.6%	140	36.2%	199	39.6%	245	36.7%	99	27.9%	323	38.5%
Diabetes	413	31.0%	1746	30.9%	115	29.7%	135	26.8%	187	28.0%	87	24.5%	270	32.1%
Obesity	358	26.9%	1286	22.8%	117	30.2%	121	24.1%	165	24.7%	88	24.8%	191	22.7%
Cancer	420	31.5%	666	11.8%	78	20.2%	116	23.1%	186	27.9%	82	23.1%	213	25.4%
Chronic renal disease	266	20.0%	627	11.1%	57	14.7%	85	16.9%	139	20.8%	51	14.4%	142	16.9%
Chronic pulmonary disease	209	15.7%	535	9.5%	55	14.2%	82	16.3%	110	16.5%	34	9.6%	140	16.7%
Neurological disease	102	7.7%	301	5.3%	16	4.1%	60	11.9%	75	11.2%	26	7.3%	59	7.0%
Chronic liver disease	130	9.8%	352	6.2%	21	5.4%	31	6.2%	52	7.8%	22	6.2%	59	7.0%
ICU specific care supports (*n*, %)														
Central venous catheter	695	52.1%	2700	47.8%	197	50.9%	284	56.5%	322	48.3%	161	45.4%	327	38.9%
Continuous hemodynamic monitoring	799	59.9%	2726	48.2%	198	51.2%	318	63.2%	368	55.2%	144	40.6%	348	41.4%
Vasoactive treatment*	746	56.0%	3234	57.2%	243	62.8%	315	62.6%	369	55.3%	192	54.1%	464	55.2%
Renal replacement therapy	212	15.9%	1007	17.8%	50	12.9%	57	11.3%	77	11.5%	46	13.0%	98	11.7%
ECMO	52	3.9%	306	5.4%	10	2.6%	7	1.4%	17	2.5%	20	5.6%	34	4.0%
Mechanical ventilation** (*n*, %)														
Non invasive/high flow oxygenotherapy	696	52.2%	2450	43.4%	229	59.2%	229	45.5%	339	50.8%	99	27.9%	463	55.1%
Invasive	849	63.7%	3823	67.7%	278	71.8%	334	66.4%	419	62.8%	242	68.2%	530	63.1%
Invasive with prone position	389	29.2%	2158	38.2%	143	37%	143	28.4%	224	33.6%	137	38.6%	302	36.0%
Death (n, %)	406	30.5%	1894	33.5%	98	25.3%	93	18.5%	151	22.6%	80	22.5%	208	24.8%

*Dobutamine, dopamine, epinephrine, norepinephrine

**One patient can have both invasive and non-invasive mechanical ventilation during their ICU stay

The factors significantly associated with the case fatality in the adjusted analyses are presented in Table 2 (all the variables initially tested are presented in Additional file 2: Table S1; all the variables ultimately included in the final model are presented in Table 2).

**Table 2:** Factors associated with case fatality in COVID-19 patients admitted in the ICU in France, March, 1st to May, 31st 2020, analyzed by logistic regression model

	Univariate	Multivariate		
	*N* = 14,513	*N* = 14,232		
	*p*	Adjusted OR	95% CI	*p*
*Patient level*				
Age				
< 65 y–o	< .0001	Ref		
65–79 y–o		2.5	[2.3–2.8]	< .0001
≥ 80 y–o		10.2	[8.7–12]	< .0001
Sex				
Female	0.0009	1.1	[1–1.2]	0.16
SAPS II*				
< 30	< .0001	Ref		
[30–40]		1.4	[1.3–1.6]	< .0001
≥ 40		2.3	[2.1–2.6]	< .0001
Comorbid conditions				
0	< .0001	Ref		
1		1.0	[0.9–1.2]	0.9
2		1.0	[0.9–1.1]	1.0
≥ 3		1.2	[1.0–1.3]	0.01
ICU specific care supports				
Invasive ventilation	< .0001	1.7	[1.5–1.9]	< .0001
Vasoactive treatment**	< .0001	1.7	[1.5–1.9]	< .0001
Renal replacement therapy	< .0001	2.9	[2.6–3.2]	< .0001
ECMO	< .0001	2.9	[2.4–3.5]	< .0001
*Hospital level*				
Number of ICU stays in hospital 2019, age ≥ 18 y–o***				
< 100	0.003	1.3	[1.1–1.4]	< .0001
[1000–2000]		1.0	[0.9–1.2]	0.73
≥ 2000		Ref		
*Regional level*				
Number of days with 75% or more of ICU beds occupied by COVID-19 patients				
< 10 days	< .0001	Ref		
10–19 days		1.2	[1.0–1.5]	0.03
20–29 days		1.5	[1.2–1.7]	< .0001
≥ 30 days		2.2	[1.9–2.6]	< .0001

*Missing data SAPS II *n* = 281

**Dobutamine, dopamine, epinephrine, norepinephrine

***For hospitals with several ICUs, all ICU stays were included

At the patient level, the risk of death increased with age (odds ratio [OR]: 2.5 [2.3–2.8] for patients of 65 to 79 years old and OR: 10.2 [8.7–12.0] for patients over 80 years old), SAPS II and comorbid conditions (Table 2).

At the hospital level, the previous experience of the health structure, assessed by the number of 2019 ICU hospitalizations, was significantly associated with the case fatality in the adjusted analyses. Healthcare facilities with less than 1000 ICU stays in 2019 were associated with a higher risk of death for patients admitted to ICU for COVID-19 (Table 2).

At the regional level, we observed an exposure–response relationship between ICU bed occupancy and case fatality rate: adjusted OR were 1.2 [1.0–1.5], 1.5 [1.2–1.7], 2.2 [1.9–2.6] for patients hospitalized in regions from 10–19 days up to ≥ 30 days of high occupancy rate of ICU beds, as compared with regions with less than 10 days of high bed occupancy rate of ICU beds (Table 2).

## Discussion

In this study, we found that the overall death rate of COVID-19 patients hospitalized in ICU during the first outbreak in France was 29.3%, but that masked tremendous inequalities at the regional level. We demonstrated that the burden of the COVID-19 crisis at the regional level (assessed by the ICU bed occupancy) was associated with higher COVID-19 mortality rate. We also demonstrated that the experience of the hospital structures (e.g., number of ICU hospitalizations in the previous year) was statistically associated with COVID-19 mortality rate. Our approach differed from conventional understanding of diseases based on patients’ characteristics and severity at presentation; instead, we investigated how healthcare organization and immediate logistic resources affected the death rate in critically ill COVID-19 patients. Results from our study showed that French regions that were overwhelmed by the COVID-19 sanitary crisis had a twofold increase in adjusted case fatality rate.

All levels components (patient, hospital, and region) were independently associated with mortality. At the patient’s level, expected results were observed: aging had the strongest association with lethal outcome [7, 8, 10]; multimorbidity and severity at presentation (SAPS II) were also linked with mortality [11]. At the regional level, the ICU bed occupancy rate was used as a surrogate of the importance of the sanitary crisis, and of ICU burden in particular. The majority of French regions have experienced occupancy rates superior to 100% of pre-existing bed capacity, demonstrating an extraordinary expansion of ICU beds by implementation of new ICU locations, within hospital or in pop-up hospital. For example in the *Grand Est* region, the number of critically ill COVID-19 patients was superior to the pre-existing ICU bed capacity of the region in 3 weeks. The expanse of critical care capacity during the pandemic has not been without any cost. Indeed, we observed that the risk of death increased gradually with the ICU occupancy rates at the regional level. Regions with more than 10 days with 75% or more of ICU beds occupied by COVID-19 patients had experienced an excess of mortality, independently to the other parameters. Intensive care medicine is a highly skill- and experienced-based medical specialty. One can assume that the implantation of temporary ICU structures has been a tremendous effort to limit the dramatic consequences of crude ICU bed shortage, but the quality of care that was provided in these conditions could hardly guaranty the highest standards of care. In agreement with this assumption, we observed that the importance of the experience of the hospital structures, assessed by the number of ICU patients treated yearly before the pandemic, was also an independent protective factor. At last, the cut-off threshold of 75% or more of ICU beds occupied by COVID-19 patients could hardly be considered as an operational marker, but is rather a late indicator. With median duration of mechanical ventilation and ICU stay of 13 (8–18) and 21 (13–36) [12], an important effort of anticipation should be performed to avoid reaching this level of ICU shortage.

The use of administrative hospital databases for epidemiological purpose has sometime been challenged or criticized. Strengths and weaknesses of this method has been already discussed [7, 8, 13–17]. Herein, there is an unprecedented opportunity to compare results obtained from prospective cohorts or from retrospective analysis of hospital discharge database HDD (present study or [12]). Indeed, the COVID-ICU study [12] was a multi-center, prospective cohort conducted in 138 hospitals (135 in France, 3 in Belgium and Switzerland) that studied 4,643 COVID-19 patients hospitalized in ICU during the same study period [12]. Consequently, a large proportion of patient overlap is expected with our 14,513-patient nationwide cohort generated from French HDD. The patients’ characteristics at admission were comparable whatever the methodology used, in COVID-ICU or the present study, respectively: sex ratio (26% of female vs 28%), mean age (63 years old, both study), proportion of hypertension (48% vs 49%) and diabetes mellitus (28% vs 31%), whereas obesity was different (41% vs 24.5%). Ventilation support characteristics in the two studies were: invasive mechanical ventilation during the ICU stay 80% vs 68.1%, extra-corporeal membrane oxygenation (ECMO) 8% vs 4%, respectively, in COVID-ICU or the present study. The few observed differences may be explained by a certain degree of inaccuracy that is an inherent bias of administrative hospital database. For example, there is probably an underestimation of obesity rate in our report. However, selective bias could also be generated by prospective database. For example, the use of ECMO was double in COVID-ICU prospective study compared to our report, but 56% of the patients in COVID-ICU were recruited in Paris where there is a worldwide expert center for ECMO [12]. Rather than opposing studies based on retrospective administrative data with studies based on prospective clinical databases, future challenges will probably be to implement novel complementary strategies combining the advantages of both approaches. The major interest of the HDD is the exhaustive record of all patients hospitalized during the studied period without initial selection bias, giving reliable information on healthcare in real life. Patients from all regions and all healthcare structures were included, and not only health structures involved in clinical research. The major interest of prospective clinical database is to provide a granularity that is indispensable to further decipher these observations.

This study had limitations. Due to the cross-sectional study design, exposure and outcome are simultaneously assessed. Thus, we cannot establish a true cause-and-effect relationship without longitudinal data. We assumed that discharge from the hospital was not a competing event for death because we, and others, observed that the death in COVID-19 critical patients occurred almost exclusively in ICU or in ward following ICU-discharge, and rarely after hospital discharge [9, 12]. Regarding the choice of the statistical methods, it is difficult to clearly identify the best method between multivariable methods and propensity scores to adjust for confounders in non-randomized studies [18]. Both standard multivariable methods and propensity scores have key limitations, and none is able to take into account unknown confounders. Debate persists on the real usefulness of propensity scores in comparison to standard multivariable approaches such as logistic regression [18, 19], but it is important to keep in mind that in many cases both approaches provide similar results [20, 21]. The use of propensity score with matching could have been used here and may have reduced errors in the estimation of the effect of the confounders on the outcome. However, the propensity score empirical coverage probability decreased after eight or more events per confounder [19] and the logistic regression seems to be a relevant choice when there are at least eight events per confounder. Because there was similar follow-up, no censored data and important number of events per covariate, we preferred to use a commonly used method, easy to understand by non-specialized readers, and to carefully report all available information (Additional file 2: Table S1 for bivariate analysis, Table 2 for logistic regression model). Next, nurse/bed ratio might have been an important factor to take into consideration but incoherencies were found in the declarative data between the number of full-time equivalents and the number of persons. We preferred not to use this information. Finally, the reliability of national health administrative systems for epidemiology purpose may vary according to case definition and could generate false positive diagnosis, whereas false-negative rate remained relatively low whatever the definition [13, 15, 22]. However, the implementation of straightforward coding for COVID-19 limited the risk of information bias. Another limitation is the potential for biases related to under-detection or misclassification, particularly for comorbidities (as discussed earlier for obesity rate). However, a good agreement between patients’ characteristics in the concomitant prospective cohort [12] was observed. For some variables, information is not recorded when there is no direct impact on patient care during hospitalization (e.g., tobacco use). Overall, rigorous acknowledgment of strength and limitation of HDD are critical to provide epidemiological data. In the context of the COVID-19 sanitary crisis, HDD-based surveillance has been promoted as a cost-effective method for healthcare service studies with real life dynamic approach, as previously demonstrated [5, 23, 24].

In conclusion, we studied critically ill COVID-19 patients during the first French outbreak and observed significant differences in adjusted case fatality rate between French regions. The regions with the highest burden of care in ICU were associated with up to 2.2-fold increase of death rate.

## Supplementary Information

**Additional file 1.** ICD-10 diagnosis codes.

**Additional file 2.** Bivariate analysis of case fatality in COVID-19 patients admitted in the ICU in France, March–May 2020.

## Data Availability

Restrictions apply to the availability of these data and so are not publicly available. However, data are available from the authors upon reasonable request and with the permission of the institution.

## Abbreviations

ATIH: *Agence Technique de l’Information sur l’Hospitalisation*
HDD: Hospital discharge database
ICU: Intensive care unit
PMSI: *Programme de Médicalisation des Systèmes d’Information*
SAE: *Statistique Annuelle des Etablissements*
SAPS II: Simplified Acute Physiology Score II
SD: Standard deviation
