# Supplementary material for: Case fatality inequalities of critically ill COVID-19 patients according to patient-, hospital- and region-related factors: a French nationwide study
Source: Ann Intensive Care. 2021 Aug 19;11:127. doi: 10.1186/s13613-021-00915-4 (PMC8375279; doi:10.1186/s13613-021-00915-4)
Supplement: Supplementary file 1 — Additional file 1. ICD-10 diagnosis codes. [file 13613_2021_915_MOESM1_ESM.docx]

**Guillon et al. C**ase fatality inequalities of critically-ill covid-19 patients according to patient-, hospital- and region-related factors: a French nationwide

**Supplementary material - Methods**

# ICD-10 codes for COVID-19

U0710 - COVID-19, respiratory presentation, identified virus

U0711 - COVID-19, respiratory presentation, virus not identified

U0712 - COVID-19, asymptomatic SARS-CoV-2, identified virus

U0714 - COVID-19, other clinical presentations, identified virus

U0715 - COVID-19, other clinical presentations, virus not identified

# ICD-10 codes for comorbidities

**HYPERTENSION:**

I10.x - Essential (primary) hypertension

I11.x - Hypertensive heart disease

I12.x - Hypertensive renal disease

I13.x - Hypertensive heart and renal disease

I15.x - Secondary hypertension

**CHRONIC HEART DISEASES:**

- ISCHEMIC HEART DISEASE

I20.x - Angina pectoris

I25.x - Chronic ischemic heart disease

Z95.5 - Presence of coronary angioplasty implant and graft

- CARDIAC, ARRHYTHMIA

I44.1 - Atrioventricular block, second degree

I44.2 - Atrioventricular block, complete

I44.3 - Other and unspecified atrioventricular block

I45.6 - Pre-excitation syndrome

I45.9 - Conduction disorder, unspecified

I47.x - Paroxysmal tachycardia

I49.x - Other cardiac arrhythmias

R00.0 - Tachycardia, unspecified

R00.1 - Bradycardia, unspecified

R00.8 - Other and unspecified abnormalities of heart beat

Z95.0 - Tachycardia, unspecified

- ATRIAL FIBRILLATION

I48.x - Atrial fibrillation and flutter

4 - CHRONIC HEART FAILURE

I11.0 - Hypertensive heart disease with (congestive) heart failure

I13.0 - Hypertensive heart and renal disease with (congestive) heart failure

I13.2 - Hypertensive heart and renal disease with both (congestive) heart failure and renal failure

I42.0 - Dilated cardiomyopathy

I42.5 - Other restrictive cardiomyopathy, Constrictive cardiomyopathy.

I42.6 - Alcoholic cardiomyopathy

I42.7 - Cardiomyopathy due to drugs and other external agents

I42.8 - Other cardiomyopathies

I42.9 - Cardiomyopathy, unspecified; Cardiomyopathy.

I43.x - Cardiomyopathy in diseases classified elsewhere

- VALVULAR DISEASE

I05.x - Rheumatic mitral valve diseases

I06.x - Rheumatic aortic valve diseases

I07.x - Rheumatic tricuspid valve diseases

I08.x - Multiple valve diseases

I34.x - Nonrheumatic mitral valve disorders

I35.x - Nonrheumatic aortic valve disorders

I36.x - Nonrheumatic tricuspid valve disorders

I37.x - Pulmonary valve disorders

I38.x - Endocarditis, valve unspecified

I39.x - Endocarditis and heart valve disorders in diseases classified elsewhere

Q23.0 - Congenital stenosis of aortic valve

Q23.1 - Congenital insufficiency of aortic valve

Q23.2 - Congenital mitral stenosis

Q23.3 - Congenital mitral insufficiency

Z95.2 - Presence of prosthetic heart valve

Z95.3 - Presence of xenogenic heart valve

Z95.4 - Presence of other heart-valve replacement

**DIABETES:**

E10.0 to E10.9 - Insulin-dependent diabetes mellitus

E11.0 to E11.9 - Non-insulin-dependent diabetes mellitus

E12.0 to E12.9 - Malnutrition-related diabetes mellitus

E13.0 to E13.9 - Other specified diabetes mellitus

E14.0 to E14.9 - Unspecified diabetes mellitus

**OBESITY:**

E66.04 Obesity due to excess calories in adults with BMI in [30-35[ or obesity due to excess calories in children

E66.05 Obesity due to excess calories in adults with BMI in [35-40[

E66.06 Obesity due to excess calories in adults with BMI in [40-50[

E66.07 Obesity due to excess calories in adults with BMI ≥ 50

E66.09 Obesity due to excess calories in adults with BMI unspecified

E66.14 Drug-induced obesity in adults with BMI in [30-35[ or drug-induced obesity in children

E66.15 Drug-induced obesity in adults with BMI in [35-40[

E66.16 Drug-induced obesity in adults with BMI in [40-50[

E66.17 Drug-induced obesity in adults with BMI ≥ 50

E66.19 Drug-induced obesity in adults with BMI unspecified

E66.24 Extreme obesity with alveolar hypoventilation in adults with BMI in [30-35[ or Extreme obesity with alveolar hypoventilation in children

E66.25 Extreme obesity with alveolar hypoventilation in adults with BMI in [35-40[

E66.26 Extreme obesity with alveolar hypoventilation in adults with BMI in [40-50[

E66.27 Extreme obesity with alveolar hypoventilation in adults with BMI ≥ 50

E66.29 Extreme obesity with alveolar hypoventilation in adults with BMI unspecified

E66.84 Other obesity in adults with BMI in [30-35[ or other obesity in children

E66.85 Other obesity in adults with BMI in [35-40[

E66.86 Other obesity in adults with BMI in [40-50[

E66.87 Other obesity in adults with BMI ≥ 50

E66.89 Other obesity in adults with BMI unspecified

E66.94 Obesity unspecified in adults with BMI in [30-35[ or obesity unspecified in children

E66.95 Obesity unspecified in adults with BMI in [35-40[

E66.96 Obesity unspecified in adults with BMI in [40-50[

E66.97 Obesity unspecified in adults with BMI ≥ 50

E66.99 Obesity unspecified in adults with BMI unspecified

**CHRONIC PULMONARY DISEASES:**

- CHRONIC OBSTRUCTIVE PULMONARY DISEASE

J43.x - Emphysema

J44.x - Other chronic obstructive pulmonary disease

- PULMONARY CIRCULATION DISORDERS

I27.x - Primary pulmonary hypertension

I28.8 - Other specified diseases of pulmonary vessels

I28.9 - Disease of pulmonary vessels, unspecified

- CHRONIC RESPIRATORY FAILURE

J96.1 - Chronic respiratory failure

**CANCER:**

- ANY MALIGNANCY, INCLUDING LYMPHOMA AND LEUKEMIA.

C00.x to C75.x - Malignant neoplasms, stated or presumed to be primary, of specified sites, except of lymphoid, hematopoietic and related tissue

C76.x - Malignant neoplasm of other and ill-defined sites

C80.x - Malignant neoplasm, without specification of site

C81.x to C96.x - Malignant neoplasms, stated or presumed to be primary, of lymphoid, hematopoietic and related tissue

C97.x - Malignant neoplasms of independent (primary) multiple sites

Z85.x - Personal history of malignant neoplasm

Z86.0 - Personal history of other neoplasms

- METASTATIC SOLID TUMOR

C77.x - Secondary and unspecified malignant neoplasm of lymph nodes

C78.x - Secondary malignant neoplasm of respiratory and digestive organs

C79.x - Secondary malignant neoplasm of other and unspecified sites

**CHRONIC RENAL DISEASES:**

- PREADMISSION DIALYSIS

Z99.2 - Dependence on renal dialysis

Z49.1 - Extracorporeal dialysis

Z49.2 - Other dialysis

- RENAL FAILURE

I12.0 - Hypertensive renal disease with renal failure

I13.1 - Hypertensive heart and renal disease with (congestive) heart failure

N18.1 - Chronic kidney disease, stage 1

N18.2 - Chronic kidney disease, stage 2

N18.3 - Chronic kidney disease, stage 3

N18.4 - Chronic kidney disease, stage 4

N18.5 - Chronic kidney disease, stage 5

N18.9 - Chronic kidney disease, unspecified

N19.x - Unspecified kidney failure

**NEUROLOGICAL DISEASES:**

- CEREBROVASCULAR DISEASE

G45.x - Transient cerebral ischaemic attacks and related syndromes

G46.x - Vascular syndromes of brain in cerebrovascular diseases

I69.x - Sequelae of cerebrovascular disease

– HEMIPLEGIA, PARAPLEGIA OR PARALYTIC SYNDROME

G04.1 - Tropical spastic paraplegia

G11.4 - Hereditary spastic paraplegia

G80.1 - Spastic diplegic cerebral palsy

G80.2 - Spastic hemiplegic cerebral palsy

G81.x - Hemiplegia

G82.x - Paraplegia and tetraplegia

G83.0 - Diplegia of upper limbs

G83.1 - Monoplegia of lower limb

G83.2 - Monoplegia of upper limb

G83.3 - Monoplegia, unspecified

G83.4 - Cauda equina syndrome

G83.9 - Paralytic syndrome, unspecified

**CHRONIC LIVER DISEASES:**

B18.x - Chronic viral hepatitis

I85.x - Oesophageal varices

I86.4 - Gastric varices

I98.2 - Esophageal varices without bleeding in diseases classified elsewhere

K70.x - Alcoholic liver disease

K71.1 - Toxic liver disease with hepatic necrosis

K71.3 - Toxic liver disease with chronic persistent hepatitis

K71.4 - Toxic liver disease with chronic lobular hepatitis

K71.5 - Toxic liver disease with chronic active hepatitis

K71.7 - Toxic liver disease with fibrosis and cirrhosis of liver

K72.x - Hepatic failure, not elsewhere classified

K73.x - Chronic hepatitis, not elsewhere classified

K74.x - Fibrosis and cirrhosis of liver

K76.x - Other diseases of liver

Z94.4 - Liver transplant status
