# Supplementary material for: Case fatality inequalities of critically ill COVID-19 patients according to patient-, hospital- and region-related factors: a French nationwide study
Source: Ann Intensive Care. 2021 Aug 19;11:127. doi: 10.1186/s13613-021-00915-4 (PMC8375279; doi:10.1186/s13613-021-00915-4)
Supplement: Supplementary file 2 — Additional file 2. Bivariate analysis of case fatality in COVID-19 patients admitted in the ICU in France, March–May 2020. [file 13613_2021_915_MOESM2_ESM.docx]

**Guillon et al. C**ase fatality inequalities of critically-ill covid-19 patients according to patient-, hospital- and region-related factors: a French nationwide

**Supplementary material - Results**

**Table S1.** Bivariate analysis of case fatality in COVID-19 patients admitted in the ICU in France, March-May 2020

|  | | |  | | **TOTAL** | | | | | **Death** | | | | **p** | | |  |  |  |
| --- | --- | --- | --- | --- | --- | --- | --- | --- | --- | --- | --- | --- | --- | --- | --- | --- | --- | --- | --- |
|  | | |  | | **(n=14,513- 100%)** | | | | | **(n=4,256 - 29.3%)** | | | |  |  |  |  |  |  |
|  | | |  | | N | | | | | N | | % | |  |  |  |  |  |  |
| ***Patient level*** | | | | | | | | | | |  | |  | | |  | |  |  |
| **Age** | < 65 y-o | | | | | 7,145 | | 1,294 | | | 18.1% | | | <.0001 | | |  |  |  |
|  | 65-79 y-o | | | | | 6,280 | | 2,328 | | | 37.1% | | |  |  |  |  |  |  |
|  | ≥ 80 y-o | | | | | 1,088 | | 634 | | | 58.3% | | |  |  |  |  |  |  |
| **Sexe** | Male | | | | | 10,377 | | 3,125 | | | 30.1% | | | 0.001 | | |  |  |  |
|  | Female | | | | | 4,136 | | 1,131 | | | 27.3% | | |  |  |  |  |  |  |
| **SAPS II*** | mean | | | | | 39.47 | | 48.18 | | |  | | | <.0001 | | |  |  |  |
|  | < 30 | | | | | 4,397 | | 572 | | | 13.0% | | | <.0001 | | |  |  |  |
|  | [30-40[ | | | | | 3,752 | | 961 | | | 25.6% | | |  |  |  |  |  |  |
|  | ≥ 40 | | | | | 6,083 | | 2,656 | | | 43.7% | | |  |  |  |  |  |  |
| **Comorbid condition** | At least one comorbidity | | | | | 11,739 | | 3,641 | | | 31.0% | | | <.0001 | | |  |  |  |
|  | High blood pressure | | | | | 7,131 | | 2,268 | | | 31.8% | | | <.0001 | | |  |  |  |
|  | Chronic heart disease | | | | | 4,971 | | 1,777 | | | 35.7% | | | <.0001 | | |  |  |  |
|  | Diabetes | | | | | 4,487 | | 1,538 | | | 34.3% | | | <.0001 | | |  |  |  |
|  | Obesity | | | | | 3,549 | | 903 | | | 25.4% | | | <.0001 | | |  |  |  |
|  | Chronic pulmonary disease | | | | | 1,913 | | 731 | | | 38.2% | | | <.0001 | | |  |  |  |
|  | Chronic liver disease | | | | | 1,046 | | 440 | | | 42.1% | | | <.0001 | | |  |  |  |
|  | Cancer | | | | | 3,078 | | 1,094 | | | 35.5% | | | <.0001 | | |  |  |  |
|  | Chronic renal disease | | | | | 2,352 | | 936 | | | 39.8% | | | <.0001 | | |  |  |  |
|  | Neurological disease | | | | | 1,120 | | 273 | | | 24.4% | | | 0.0002 | | |  |  |  |
| **Number of comorbid conditions** | 0 | | | | | 2,774 | | 615 | | | 22.2% | | | <.0001 | | |  |  |  |
|  | 1 | | | | | 3,507 | | 922 | | | 26.3% | | |  |  |  |  |  |  |
|  | 2 | | | | | 3,284 | | 972 | | | 29.6% | | |  |  |  |  |  |  |
|  | ≥3 | | | | | 4,948 | | 1,747 | | | 35.3% | | |  |  |  |  |  |  |
| **Specific Care Supports** | Central venous catheter | | | | | 7,407 | | 2,588 | | | 34.9% | | | <.0001 | | |  |  |  |
|  | Arterial catheter | | | | | 7,168 | | 2,339 | | | 32.6% | | | <.0001 | | |  |  |  |
|  | Continuous hemodynamic monitoring | | | | | 7,601 | | 2,485 | | | 32.7% | | | <.0001 | | |  |  |  |
|  | Vasoactive treatment* | | | | | 8,528 | | 3,272 | | | 38.4% | | | <.0001 | | |  |  |  |
|  | Non invasive ventilation / high flow oxygenotherapy | | | | | 6,546 | | 1,500 | | | 22.9% | | | <.0001 | | |  |  |  |
|  | Invasive ventilation | | | | | 9,885 | | 3,537 | | | 35.8% | | | <.0001 | | |  |  |  |
|  | Invasive ventilation with prone position | | | | | 5,534 | | 2,099 | | | 37.9% | | | <.0001 | | |  |  |  |
|  | Renal replacement therapy | | | | | 2,165 | | 1,247 | | | 57.6% | | | <.0001 | | |  |  |  |
|  | ECMO | | | | | 587 | | 300 | | | 51.1% | | | <.0001 | | |  |  |  |
| ***Hospital level*** | | | | | | | | | | |  | |  | | |  | |  |  |
| **Type of hospital** | Teaching or regional hospital | | | | | | 5,930 | | 1,717 | | | 29.0% | | | 0.41 | |  |  |  |
|  | Local public hospital, private or mixed facility | | | | | | 8,583 | | 2,539 | | | 29.6% | | |  |  |  |  |  |
| **Number of ICU stays in hospital 2019***** | <1,000 stays | | | | | | 7,984 | | 2,433 | | | 30.5% | | | 0.003 | |  |  |  |
|  | [1,000-2,000[ stays | | | | | | 4,126 | | 1,144 | | | 27.7% | | |  |  |  |  |  |
|  | ≥2,000 stays | | | | | | 2,403 | | 679 | | | 28.3% | | |  |  |  |  |  |
| **Number of physicians in ICU / number of ICU beds in hospital 2019** | <0.5 | | | | | | 8,032 | | 2,361 | | | 29.4% | | | 0.98 | |  |  |  |
|  | [0.5-1[ | | | | | | 5,425 | | 1,586 | | | 29.2% | | |  |  |  |  |  |
|  | ≥1 | | | | | | 1,056 | | 309 | | | 29.3% | | |  |  |  |  |  |
| ***Regional level*** | | | | | | | | | | |  | |  | | |  | |  |  |
| **Location of the patient** | | *Auvergne-Rhône-Alpes* | | 1,358 | | | | | 347 | | | 25.6% | | | <.0001 | |  |  |  |
|  |  | *Bourgogne-Franche-Comté* | | 580 | | | | | 175 | | | 30.2% | | |  |  |  |  |  |
|  |  | *Bretagne* | | 256 | | | | | 45 | | | 17.6% | | |  |  |  |  |  |
|  |  | *Centre-Val de Loire* | | 410 | | | | | 87 | | | 21.2% | | |  |  |  |  |  |
|  |  | *Corse* | | 44 | | | | | 12 | | | 27.3% | | |  |  |  |  |  |
|  |  | *Grand Est* | | 2,130 | | | | | 660 | | | 31.0% | | |  |  |  |  |  |
|  |  | *Hauts-de-France* | | 1,333 | | | | | 406 | | | 30.5% | | |  |  |  |  |  |
|  |  | *Ile-de-France* | | 5,650 | | | | | 1,894 | | | 33.5% | | |  |  |  |  |  |
|  |  | *Normandie* | | 387 | | | | | 98 | | | 25.3% | | |  |  |  |  |  |
|  |  | *Nouvelle-Aquitaine* | | 503 | | | | | 93 | | | 18.5% | | |  |  |  |  |  |
|  |  | *Occitanie* | | 667 | | | | | 151 | | | 22.6% | | |  |  |  |  |  |
|  |  | *Pays de la Loire* | | 355 | | | | | 80 | | | 22.5% | | |  |  |  |  |  |
|  |  | *Provence-Alpes-Côte d'Azur* | | 840 | | | | | 208 | | | 24.8% | | |  |  |  |  |  |
| **Number of days over 75% of occupation of ICU beds by COVID-19 patients in the region** | | <10 days | | 1,426 | | | | | 289 | | | 20.3% | | | <.0001 | |  |  |  |
|  |  | 10-19 days | | 1,992 | | | | | 473 | | | 23.7% | | |  |  |  |  |  |
|  |  | 20-29 days | | 2,735 | | | | | 765 | | | 28.0% | | |  |  |  |  |  |
|  |  | ≥30 | | 8,360 | | | | | 2,729 | | | 32.6% | | |  |  |  |  |  |
| ** missing data SAPS II n=281* | | | | | | | | | | | | | | | | | | | |
| *** dobutamin, dopamin, epinephrine, norepinephrine* | | | | | | | | | | | | | | | | | | | |
| **** for hospitals with several ICUs, all ICU stays were included* | | | | | | | | | | | | | | | | | | | |
